# What Makes Us React to the Abuse of Pets, Protected Animals, and Farm Animals: The Role of Attitudes, Norms, and Moral Obligation

**DOI:** 10.3390/ani15223339

**Published:** 2025-11-19

**Authors:** Cristina Ruiz, Andrea Vera, Christian Rosales, Ana M. Martín

**Affiliations:** Departamento de Psicología Cognitiva, Social y Organizacional, Universidad de la Laguna, 38200 San Cristóbal de La Laguna, Spain; cruizpa@ull.edu.es (C.R.); averasua@ull.edu.es (A.V.); crosales@ull.edu.es (C.R.)

**Keywords:** animal abuse, personal norms, social norms, moral obligation, speciesism, attitudes towards animals, environmental laws, animal welfare laws

## Abstract

This study tests a theoretical model explaining reactions to animal abuse in terms of attitudes, norms, and moral obligation, based on research concerning pro-environmental and anti-ecological behavior, as offenses against animals have been considered environmental crimes in legal terms. The sample consisted of 624 people from the general population, aged 18 to 93 (64.1% female), randomly assigned one of three versions of the same scenario of abuse, differing in the category of animal (protected/pet/farm). Participants were requested to complete a questionnaire that included items about the observed variables (descriptive social norm) and latent variables (injunctive social norm, personal norm, moral obligation, attitude toward animals, speciesism, and reaction to animal abuse). The resulting model obtained appropriate fit indices (RMSEA = 0.054; CFI = 0.917) and a high percentage of explained variance of reaction (77%) and confirmed the expectation that moral obligation is the strongest predictor of reactions to animal abuse and activates the personal norm. Personal norm is predicted by attitudes toward animals and the injunctive social norm, which depends on the descriptive social norm. Speciesism was excluded from the model due to its negative covariance with attitudes toward animals and to provide a better-fitting model. The results are discussed in terms of how the human–animal relationship is mediated by the role played in animal categorization, not only by their characteristics, but also by the instrumentality attributed to them socially and culturally.

## Simple Summary

This study uses structural equation analysis to test a model explaining reactions to animal abuse in terms of attitudes, norms, and moral obligation. This model was based on research concerning pro-environmental and anti-ecological behavior, as offenses against animals are considered environmental crimes in legal terms. The sample consisted of 624 people from the general population, aged 18 to 93, 64.1% of whom were female. Participants were recruited by the snowball technique using Students of Social Work and Psychology degrees, who were asked to contact people of different genders, ages, and areas of residence and spread the link to access the questionnaire in exchange for extra points in some subjects. They were randomly assigned one of three versions of the same scenario, differing in the category of abused animal (protected/pet/farm), to be rated on several items. These items served as indicators of the observed variables (descriptive social norm) and of the latent variables (injunctive social norm, personal norm, moral obligation, attitude toward animals, speciesism, and reaction to animal abuse). The model was tested using SEM, obtaining appropriate fit indices (RMSEA = 0.054; CFI = 0.917) and a high percentage of explained variance of reaction (77%). The results confirmed the expectation that moral obligation is the strongest predictor of reactions to animal abuse and activates the personal norm. Personal norm is predicted by attitudes toward animals and the injunctive social norm, which depends on the descriptive social norm. The results are discussed in terms of how the human–animal relationship is mediated by the role played in animal categorization, not only by their characteristics, but also by the instrumentality attributed to them socially and culturally.

## 1. Introduction

There has been a notable rise in interest in animal abuse among the general population in recent decades (e.g., https://www.dsca.gob.es/es/derechos-sociales/derechos-animales/estudios (accessed on 30 October 2025); [1]). This phenomenon has been associated with growing concern about responsible environmental behavior, as well as changes in lifestyle and dietary options (e.g., [2]). The term “responsible environmental behavior” [3] is used interchangeably with “environmentally significant behavior” [4] or “pro-environmental behavior” [5] to refer to behaviors aimed at protecting the environment. Concurrently, legislators have proposed laws to criminalize animal abuse and to enhance public awareness of the need for respectful and considerate behavior toward animals (see Section 2.1). To facilitate the analysis of these changes, research has been conducted across a range of disciplines with the aim of reducing the incidence of animal abuse (e.g., [6,7,8]). A fundamental objective of psychological research is to elucidate the underlying causes of behavior, as this knowledge is essential for the development of effective interventions. Consequently, a considerable body of research has been conducted with the aim of establishing the extent to which attitudes, norms and motivations, and other psychosocial variables precede behavior in a range of fields [9,10,11,12,13]. In the case of animal abuse, it is important to identify not only the causes of those who perpetrate it but also those who react to the abuse inflicted by others. This understanding is essential for the changes that society must make to address this issue. The present study aims to ascertain the extent to which the reaction to animal abuse can be explained by attitudes toward animals, personal and social norms, and moral obligation. More specifically, a theoretical model formulated on the basis of previous research will be tested. This research has provided evidence showing that moral obligation is a direct predictor of behavior, and it is activated through the social and personal norms that people adopt as their own, as well as the attitudes they hold toward the object of behavior.

Research has shown that attitudes toward animals are important for human–animal relationships and the support of social movements that advocate for animal rights [14]. Attitude is defined in terms of Eagly & Chaiken [15] as “an individual’s propensity to evaluate a particular entity with some degree of favorability or unfavorability” (p. 583)—see also Conner & Norman [10] for a recent review on attitudes, intentions, and behavior change. People with more positive attitudes toward animals show greater concern about animal abuse and agree with more severe punishments for animal abusers than those who have less positive attitudes to animals [16]. These attitudes relate negatively to speciesism, the belief that humans are intrinsically more valuable than animals, or that some animals are more valuable than others [17]. Considering animal species inferior to humans may be behind animal abuse or cruel behavior [18]. Speciesism may also be the reason for the lack of reaction to the abuse inflicted by others or the social and moral acceptance of the abuse of certain species of animals [19,20,21]. The category of animal (pets, wild species, …) has also been found to moderate these relationships [22].

However, attitudes are not sufficient to explain behavior. According to the theory of planned action [23], widely used to analyze pro-environmental behavior [24], norms must also be included as explanatory variables to predict an individual’s intention to perform a behavior (herein, behavioral intention). Integrating norms and attitudes in the same model has been shown in research to provide superior explanatory power for the intention to act on environmental crime, justification for actions against the environment, or the intention for pro-environmental behavior in rural contexts [25,26,27].

Norms are proposed as the framework which guides people’s pro-environmental behavior and can be personal, if they are the individual’s own convictions, or social, insofar as they are group constructions [1]. Social norms have been conceptualized as descriptive, injunctive, and subjective. The activation of one or another type of social norm generates different behaviors [28]. Descriptive social norms involve perceptions of which behaviors most people perform, and they are motivated by the fact that they provide evidence of what is effective and adaptive. Injunctive social norms involve perceptions of which behaviors are typically approved or disapproved by most people [29]. Subjective social norms refer only to the approval or disapproval of significant others [30,31]. Both types of norms are motivated by externally administered rewards or punishments or by the anticipation of them [29,30].

**Figure 1:**
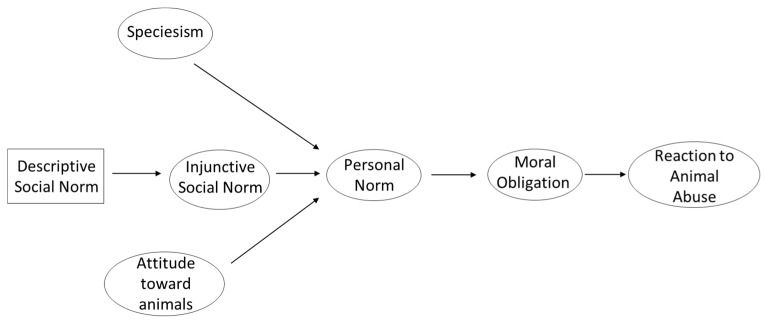
Initial explanatory model of the reaction to the animal abuse in terms of attitudes, norms and the moral obligation.

According to Schwartz’s [32] model of norm activation, personal norm has the greatest predictive power over behavioral intention, although it may be regulated by other factors, such as awareness of consequences or responsibility denial. In contrast, other authors posit that social norms, whether injunctive or descriptive, precede the personal norm along the continuum of increasing levels of internalization and integration into the self, as proposed by Thørgensen [30,33]. Typically, a social norm ends up being internalized into a personal norm through a process of self-categorization and identification of the individual with the group [34,35]. Results recently found in a meta-analysis of 572 studies on pro-environmental behavior support this continuum. Specifically, the effect of the personal norm on behavioral intention and final behavior was found to be the greatest, although this does not detract from the importance of descriptive, injunctive, and subjective social norms, which define what should be done or what most people do [33]. Although there may be indirect relationships between social norms and behavior, a direct relationship is expected to be found between descriptive and injunctive norms, as well as between these social norms and personal norms, reflecting this progressive internalization (see Figure 1).

It is expected, therefore, that the reaction to animal abuse is also directly related to personal norms and indirectly to the social norms that are salient in a given context and at a given time. These social norms will be shaped by what people in general and people who are affective referents are believed to think. Much of the research studying the relationship between social norms and behaviors toward animals has been carried out in the field of animal welfare and meat consumption and uses the theory of reasoned action to test the impact of attitudes and norms on behavioral intention [36,37]. In the cross-sectional study carried out by McGuire et al. [38] to assess the development of speciesism, considered as a negative attitude toward animals, they conclude that, in childhood, no distinction is made between animals on the basis of whether or not they can become food, and that it is the social and moral context in which individuals’ development takes place that marks, with its norms, the acceptance or rejection of animal abuse.

While attitudes and norms are the most studied variables in the field of pro-environmental behavior, recent research demonstrates the explanatory power of another variable, moral obligation. Moral obligation can be understood as the link between personal norm and behavioral intention, as it refers to the motivation to behave based on established moral norms [39]. Many studies focusing on action motivation have found that moral obligation has an impact on the behavioral intention or on the behavior itself [40].

Moral obligation has traditionally been conceptualized as the feeling that one has to act in a certain way in a situation [32]. In this sense, Deci and Ryan’s [41] self-determination theory can be very useful to measure moral obligation through different types of regulation. These authors argue that the basic needs for autonomy, competence, and relatedness regulate the internalization process of motivation, which concludes with the integration of behavior into one’s own value system and personal identity. Feelings of obligation, shame, pride, and identification are incorporated into this motivation, leading to self-determination to act. Based on this theory, Pelletier et al. [42] developed the Motivation Toward the Environment Scale (MTES), which has proven to be useful for predicting pro-environmental behavior in various situations [43,44,45]. Using the Spanish adaptation of this scale, Martín et al. [46] found that different types of self-regulation are related to the likelihood of carrying out both pro-environmental and illegal anti-environmental behaviors, in line with Thørgensen’s [30] norm internalization continuum.

The aim of this study is to test a structural model that explains the reaction one would have when witnessing a situation of animal abuse. As reflected in Figure 1, it is hypothesized that moral obligation will be the main antecedent of the intention to act, activating the personal norm. This prediction is consistent with Schwartz’s [32] norm activation theory and is supported by previous evidence [10,26]. The indicators chosen to measure the personal norm will be the moral judgment [47] and the perceived legitimacy of the law [48]. Therefore, the following is anticipated:

**H1.**  *A direct, positive, and significant impact of the moral obligation on the reaction to animal abuse*.

**H2.**  *A direct, positive, and significant impact of the personal norm on the moral obligation*.

As proposed by the focal theory of normative conduct [29], the descriptive social norm is expected to be an antecedent of the injunctive social norm and, following the theories of self-categorization [35] and group identification [34], injunctive social norm is internalized through the personal norm [10]. The injunctive social norm will be measured through indicators of subjective social norm and the prescriptive social norm [29]. Attitudes toward animals and speciesism will also influence the reaction to animal abuse [21], not directly but through personal norms [26]. The following is hypothesized:

**H3.**  *A direct, positive, and significant impact of the descriptive social norm on the injunctive social norm*.

**H4.**  *A direct, positive, and significant impact of the injunctive social norm on the personal norm*.

**H5.**  *A direct and significant impact of both the attitudes toward animals and the speciesism on the personal norm*.

This model is expected to be the same for three categories of animals in terms of the direction of the influence of some variables on others, although with some differences in their impact, considering the previous literature. These three categories are pets, farm animals, and protected animals, as stated in the legal framework described in Section 2.1. However, given the exploratory nature of this study, as it is the first to simultaneously analyze the impact of attitudes, norms, and the moral obligation on the abuse of the three categories of animals, the exact nature of these differences will not be specified.

## 2. Materials and Methods

### 2.1. The Study Setting

This study was conducted in Tenerife, the most populated and largest of the Canary Islands. Although it is the second largest among all Spanish islands in terms of surface area, it holds the highest population. Tenerife is subject to extensive environmental protection, with 48.6% of its land falling under environmental legal frameworks. The island hosts 43 officially designated natural sites notable for their biodiversity, including a significant presence of endemic plant and animal species. These protected zones encompass a range of conservation categories such as a national park, natural monuments, protected landscapes, natural and countryside parks, special and integral nature reserves, as well as areas classified as sites of special scientific interest, https://www.tenerifeon.es/en/protected-natural-areas/ (accessed on 30 October 2025). The Canary Islands constitute one of Spain’s 19 autonomous communities, where national environmental legislation applies (see below), exhibiting regulatory similarities to federal systems in other Western nations. These environmental laws govern not only the protection of wildlife and plant life but also prohibit unauthorized constructions, improper handling or disposal of hazardous or general waste, unlawful exploitation of natural resources, and any unapproved interventions or alterations in protected environments, among other infractions.

Furthermore, Spain’s status as a European Union member state places it under the legal framework established by the European Parliament, which has recently redefined the legal consideration of animals, recognizing them as sentient beings rather than mere property (https://www.europarl.europa.eu/topics/en/article/20200624STO81911/animal-welfare-and-protection-eu-laws-explained-videos/ (accessed on 30 October 2025)). This shift has prompted several EU countries, including Spain, to revise their animal welfare legislation (https://www.animal-ethics.org/la-situacion-legal-de-los-animales-en-europa/ (accessed on 30 October 2025)). Consequently, Spanish criminal law has been updated to extend its protection beyond wild fauna to encompass other animal categories. A major legislative milestone was reached in 2021 with the enactment of the Ley Orgánica 17/2021, which reformed the Civil Code, the Mortgage Act, and the Civil Procedure Act by declaring that animals are not objects but sentient beings whose interests must be safeguarded. Before this amendment, the Spanish Criminal Code had addressed animal cruelty under Title XVI, alongside other environmental crimes, and, more specifically, in Chapter III, offenses against natural resources, and in Chapter IV, offenses against flora and fauna and the natural environment (arts. 332–336; 338–340). In 2023, further advancements were made with the approval of Ley Orgánica 3/2023, which revised the Criminal Code by adding Title XVIbis offenses against animals, aimed at animals that are domestic, domesticated, or living temporarily or permanently under human control. That same year, the Spanish Parliament passed Ley 7/2023, aiming to ensure the protection and well-being of companion animals and wild animals kept in captivity.

In countries such as Germany (§90 a BGB), Austria, Switzerland, France, Portugal, and the Czech Republic, a distinct legal classification has been introduced for non-human animals, differentiating them from both “persons” and “things.” However, although legal terminology varies across jurisdictions, this categorization has been largely symbolic; in practice, animals remain subject to property law. The classification, while stating animals are not “things,” does not provide a more accurate legal representation of their nature. In contrast, the Spanish approach to animal protection and welfare appears to be among the most progressive within the EU, aligning closely with the European Parliament legislation. Nevertheless, current limitations persist within the Spanish Criminal Code, as not all animals are afforded equal legal consideration. Acts of animal cruelty are only criminalized when they occur “outside of legally regulated activities” (art. 340 bis). Moreover, Ley Orgánica 7/2023 explicitly excludes various animal categories from its scope, including those used in bullfighting, livestock animals, animals involved in experimental and scientific research (including educational use), animals in veterinary clinical studies, free-ranging wild animals, and those used in sports or professional contexts, such as hunting dogs, packs, and aids (art. 1). Public opinion in Spain also tends to differentiate between types of animals, particularly in terms of their capacity to experience pain, their familial status, and the severity of punishment deserved by abusers. These distinctions appear to be based primarily on the degree of emotional attachment people report toward different animal types [1]. Consequently, this legal and sociocultural context—marked by significant advances and lingering inconsistencies in legal status—makes Tenerife a particularly appropriate and relevant setting for investigating the study’s research questions.

### 2.2. Participants

Initially, 820 people participated in this research. To clean the data matrix, those who had not answered the entire questionnaire and/or had a high social desirability score (a score higher than 14) [49] were eliminated. The social desirability score was used to control that participants’ responses reflected their true opinions and not a desire to conform to social convention, as animal abuse may be considered a sensitive topic. The final sample consisted of 624 people, 64.1% of whom were female. The age of the participants was between 18 and 93 years old (*M* = 34.01; *SD* = 16.34). More than half of the participants stated they lived in an urban area (69.4%), 22% in a rural area, and the rest in a coastal area (8.7%). Approximately half of the participants had a university education (45.6%), 23.2% had a baccalaureate, 20% had vocational training, and only a minority stated that they had either completed Compulsory Secondary Education (4.6%) or had not completed it (4.4%). Most participants indicated that they were working (43.3%) or studying (43.1%), the rest were retired (6.9%) or unemployed (6.7%). Regarding their dietary option, 91.2% reported being omnivorous, 5.6% vegetarian, 2.1% pescetarian, and only 1% vegan.

### 2.3. Instruments

Participants were administered a questionnaire with the instruments described below in the order in which they were included.

#### 2.3.1. Scenarios Depicting Situations of Animal Abuse

Three versions of the same animal abuse scenario were developed that differed in the category of animal abused (protected/pet/farm): “Imagine you are out for a walk, and you see a person who starts hitting and kicking [category of animal] who is whimpering and wriggling around trying to run away”. Participants were randomly asked to rate one of the three types of scenarios on a series of items, grouped into the three sections described below, using an 11-point Likert-type scale from 0 = strongly disagree to 10 = strongly agree. The 11-point Likert-type scale was preferred over the 5-point scale, primarily because the former is employed in the Spanish school grading system and the participants are more accustomed to it than with the latter, which is more prevalent in Anglo-Saxon school grading systems.

#### 2.3.2. Social and Personal Norms

Three items were used to measure social norms, one as an indicator of the exogenous variable Descriptive Social Norm (DSN) and two referring to the latent variable injunctive social norm. These two items were indicators of the Subjective Social Norm (SSN) and the Prescriptive Social Norm (PSN), respectively. The latent variable Personal Norm was measured with two items relating to the indicators of Moral Judgment (MJ) and Perceived Legitimacy of the Law (PLL). These items were taken from Martín et al. [26], who based them on Cialdini et al. [29], Thørgensen [30], and Tyler [31,47,48] (see Appendix A).

#### 2.3.3. Moral Obligation

The latent variable Moral Obligation was measured with ten items. Nine items were taken from Martín et al.’s [46] Spanish adaptation of the Motivation Toward the Environment Scale [42], following Deci and Ryan’s [41] definitions of introjected, identified, and integrated regulation. Three items were used as indicators for each type of regulation, whereas the tenth was taken from the general definition of moral obligation in Schwartz’s [32] norm activation theory (see Appendix A).

#### 2.3.4. Reaction to Animal Abuse

The latent variable Reaction to Animal Abuse was measured according to behaviors commonly used in the scientific literature on prosocial behavior and involving a greater or lesser degree of personal intervention in the situation to help the abused animal. From an initial incidental sampling, a panel of judges agreed on nine behaviors as the most likely in the research context (see Appendix A).

Participants also answered two scales on Speciesism and on Attitudes toward animals and one on Social Desirability, described below.

#### 2.3.5. Speciesism Scale

The Speciesism Scale (SS) was developed by Caviola et al. [50] and consists of six items measuring the consideration that humans have a higher value than animals and that certain animals are more valuable than others (e.g., “It is morally acceptable to perform medical experiments on animals that we would not perform on any human”). On this occasion, participants answered using an 11-point Likert-type scale from 0 = strongly disagree to 10 = strongly agree. The Spanish adaptation by Suárez-Yera et al. [51] was applied. Caviola et al. [50] and Suárez-Yera et al. [51] provide evidence of the validity and internal consistency of 0.81 and 0.82, respectively.

#### 2.3.6. Animal Attitude Scale-10

The Animal Attitude Scale-10 [52] (AAS) is a shortened version of the original scale, consisting of ten items and measuring attitudes toward the use of animals by humans (e.g., “I sometimes get upset when I see wild animals in cages at zoos”). On this occasion, participants answered using an 11-point Likert-type scale from 0 = strongly disagree to 10 = strongly agree. The Spanish adaptation by Suárez-Yera et al. [51] was used. Herzog et al. [52] and Suárez-Yera et al. [51] provide evidence of the validity and internal consistency of 0.90 and 0.85, respectively.

#### 2.3.7. Social Desirability Scale

The scale of Crowne & Marlowe [53] was applied in the version adapted to Spanish and reduced by Gutiérrez et al. [49]. It consists of 18 items that measure the tendency of participants to respond in a socially appropriate way and are answered dichotomously, with 0 = False and 1 = True (e.g., “I always try to practice what I preach”). Since this tool is an index rather than a scale, the final score is obtained by adding up the positive responses. Therefore, it is not relevant to calculate its internal consistency [54]. However, Gutiérrez et al. [49] provided evidence of validity and internal consistency of this reduced scale of 0.78.

#### 2.3.8. Sociodemographic Characteristics

Participants were also asked about their gender, age, education level, employment status, place of residence (urban/rural/coastal), and type of diet.

### 2.4. Procedure

The questionnaire was administered online through the Qualtrics^XM^ platform using the snowball technique. Students enrolled in Social Work and Psychology degrees were asked to spread the link to access the questionnaire among family, friends, acquaintances, and personal social networks. They received extra points in one of their academic subjects as compensation, depending on how many questionnaires they gathered. They were asked to contact people of different genders, ages, and areas of residence to avoid the final sample being composed of only people from specific social groups, which is a potential bias of the snowball technique. The questionnaire was available for five months.

In the general instructions of the questionnaire, participants were informed that the object of the research was the study of human–animal relationships. They were guaranteed anonymity and confidentiality, as well as that their data would be exclusively used for research purposes and publication in scientific journals. They were asked to indicate their explicit consent to participate before answering the questions. They were then asked for their sociodemographic data, and their task was described. Participants were randomly asked to rate one of the three categories of animals using the Qualtrics randomizer. Equivalent distribution by gender was guaranteed by checking frequencies in the final sample. To ensure that they answered correctly in relation to each category of animal (protected/pet/farm), they were provided with several prototypical examples of the category (e.g., “…a scene is presented that describes a behavior against an animal that people usually keep as a pet (e.g., dog, cat, etc.)”). The procedure followed the ethical principles established in the Declaration of Helsinki and was approved by the Ethics Committee of the University of La Laguna (CEIBA2022-3220).

### 2.5. Design and Data Analysis

An explanatory design with latent variables was carried out [55], with the aim of testing a model to predict Reaction to Animal Abuse based on the relationship between the variables under study. The exogenous variables were Descriptive Social Norm, Attitudes toward animals, and Speciesism. The endogenous variables were Prescriptive Social Norm, Personal Norm, Moral Obligation, and Reaction to Animal Abuse. In addition, the aim was to test whether the proposed model was similar for the three categories of animals (protected, pet, and farm) under study.

Statistical analyses were carried out with the RStudio 2024.12.1-563, using the ULLRToolbox [56]. To conduct the structural equation modeling, the measurement model was first checked to confirm the relationships between the indicators and the latent variables. Then, the structural model was calculated by defining the relationships between the latent variables according to the hypothesized model using the MRL estimator, appropriate given the non-normality of the variables. To measure the goodness of fit of the proposed model to the data obtained, several statistical indicators were used in addition to χ^2^, as this tends to have a statistically significant value when sample sizes are large. The indicators chosen were the Root Mean Square Error of Approximation (RMSEA), Bentler–Bonett Non-Normed Fit Index (NNFI), Comparative Fit Index (CFI), and Tucker–Lewis Index (TLI) [57,58].

## 3. Results

To enhance the validity of the measurements required for structural equation modeling, the measurement model of the AAS and SS was first tested with confirmatory factor analysis. To do this, the results of the preliminary analyses of the data from the scales were checked against a conceptual analysis of the items based on a review of the literature. As a result, item SS5 of the SS, as well as items AAS2, AAS3, AAS4, AAS7, and AAS8 of the AAS were excluded, as the first item measured a positive attitude toward animals and the last five items measured speciesism (see Appendix A). The eliminated items had been presented in reverse order to the rest of the items on their respective original scales.

Likewise, the items used to measure the latent variable Moral Obligation were initially selected based on their reference to one of three regulatory mechanisms: introjected, identified, or integrated. However, items MO5, MO6, and MO7, which were associated with identified regulation, had to be eliminated, and the remaining items collapsed into a single internal regulation. This decision responded to the high collinearity of the items (HTMT > 0.85), especially those belonging to the identified regulation, compared to those belonging to the introjected and integrated regulations (see Appendix A). A confirmatory factor analysis (CFA) of the general (“G”) factor for moral obligation showed excellent model fit: CFI = 0.980, TLI = 0.973, RMSEA = 0.061 [0.043, 0.080]. Reliability estimates were also high (ω_Bollen = 0.912; ω_McDonald = 0.912). These decisions to eliminate items related to integrated regulation and merge the rest into a single regulation coincide with previous research by authors such as Thørgensen [30] and De Groot and Steg [59], respectively.

Finally, items RAA5, RAA7, and RAA8 used to measure the latent variable Reaction to Animal Abuse were eliminated for three different reasons. Item RAA5 (“I would record it with my mobile phone”) was dropped because participants’ responses were concentrated at the two extremes of the scale (0 = “I do not agree at all” or 10 = “I strongly agree”), leading to a misleading mean. Regarding item RAA7 (“I would throw something at the aggressor”), most participants said that they would never engage in such behavior. Item RAA8 (“I would stop to see what is going on”), originally defined as a form of non-action, was removed as participants interpreted it as a form of action but not related to the rest of the behaviors included in the scale (see Appendix A).

**Figure 2:**
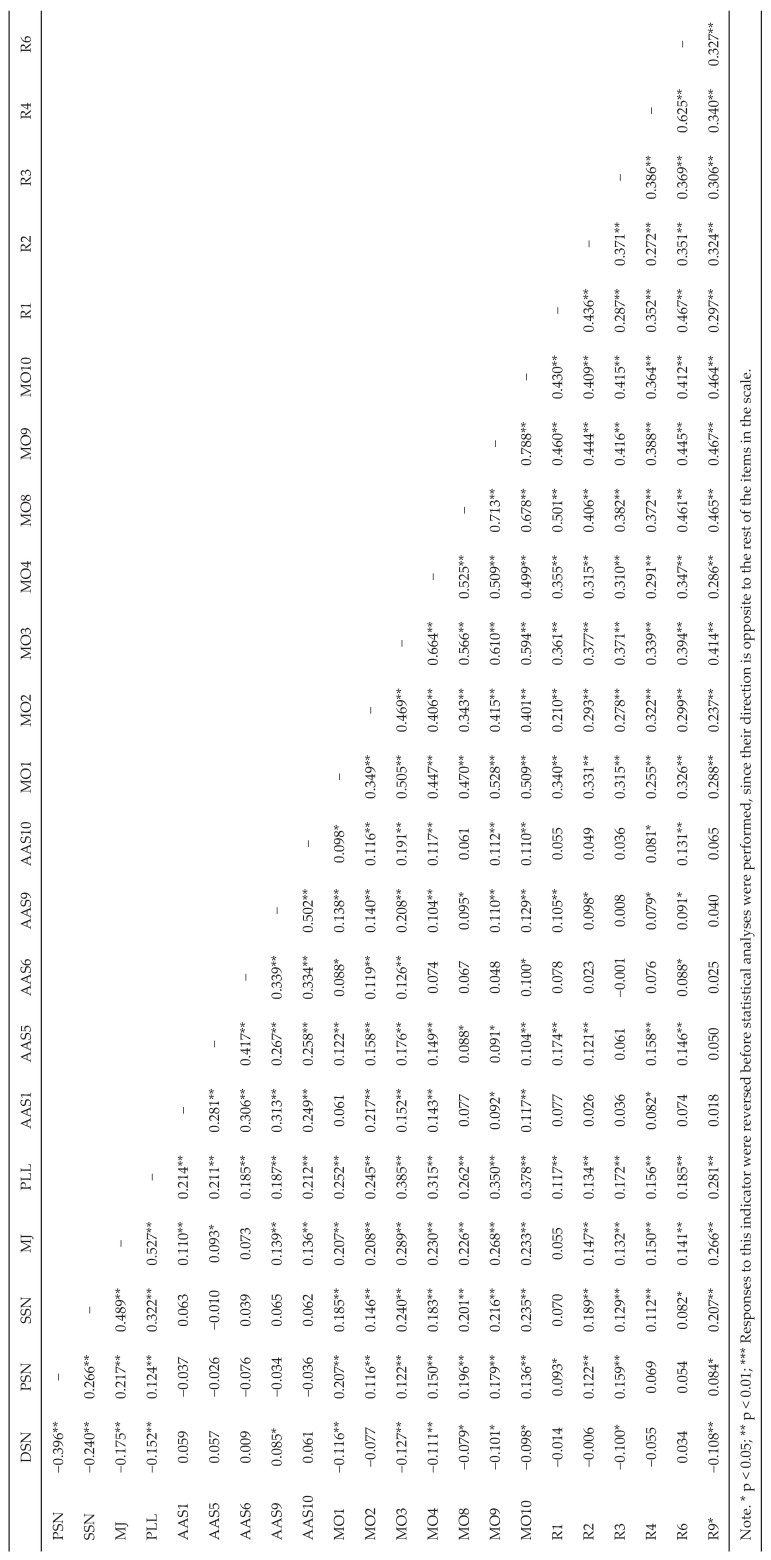
Correlations between the indicators used in the final model of the reaction to animal abuse as a function of attitudes, norms, and moral obligation.

After verifying the validity of the measurement adjustments, the proposed model was tested for the first time (Figure 1). The initial results revealed an important negative covariance (−0.402) between the latent variables Speciesism and Attitudes toward animals, which affected the explained variance of Personal Norm. To improve the fit, Speciesism was removed from the model, since a best fit was obtained with Attitudes toward animals, and recent research (e.g., [14,21]) has also opted to use AAS to measure Speciesism. Table 1 presents the means and standard deviations as well as the skewness, kurtosis, and standard error of the items corresponding to the indicators finally included in the model. The correlation matrix is in Figure 2.

The fit of the final proposed model was then analyzed using structural equation modeling. The results obtained showed that, as usual with large sample sizes, the χ^2^ was statistically significant (χ^2^ (222, *N* = 624) = 446.962, *p* < 0.001). However, the remaining indices confirmed the fit of the model to the data analyzed (RMSEA = 0.044, 90% CI [0.038, 0.050], CFI = 0.946; NNFI = 0.938; TLI = 0.938). To obtain this adjustment, the error covariances of the indicators R4 and R6 (0.432, *z* = 6.592, *p* < 0.000) of the latent variable Reaction to Animal Abuse, MO3 and MO4 (0.369, *z* = 4.530, *p* < 0.000) and MO9 and MO10 (0.313, *z* = 3.349, *p* < 0.001) of the latent variable Moral Obligation, and AAS9 and AAS10 (0.323, *z* = 4.446, *p* < 0.000) of the latent variable Attitudes toward animals had to be released.

Next, a multi-group analysis was carried out to assess whether the model was invariant in relation to the three categories of animals: protected (*n* = 199), pets (*n* = 218), and farm (*n* = 207). For this purpose, a Configural Model was estimated with no restrictions between the groups. Fit indices, although slightly lower given the smaller sample size in each group, remained within acceptable ranges (RMSEA = 0.056, 90% CI = [0.049, 0.062]; CFI = 0.916; TLI = 0.904), except for the Chi-square, which continued to be statistically significant (χ^2^ (666, *N* = 624) = 1059.156, *p* < 0.001). These results showed that the basic structure of the model is replicated for all three groups. To test for metric invariance, a second model was estimated in which factor loadings were constrained to be equal across all measurement models: χ^2^ (700) = 1084.7, *p* < 0.001, CFI = 0.917, TLI = 0.910, and RMSEA = 0.054, CI [0.048–0.060]. The comparison between the two models supported metric invariance based on the magnitude of differences across all indices: Δχ^2^ (34) = 30, *p* > 0.05, ΔCFI = 0.001, and ΔRMSEA = 0.003. Scalar invariance was tested by adding equality constraints on the intercepts of the observed indicators to the previous metric model. The resulting model yielded χ^2^ (734) = 1157.2, *p* < 0.001, CFI = 0.909, TLI = 0.906, and RMSEA = 0.055, CI [0.049–0.061]. The comparison indicated scalar invariance based on ΔCFI = 0.008, ΔTLI = 0.004, and ΔRMSEA = 0.002, as these differences were well below the recommended thresholds of 0.01 and 0.015 [60,61], although the Δχ^2^ (34) = 74.1, *p* < 0.001 statistic was significant (see Table 2).

**Figure 3:**
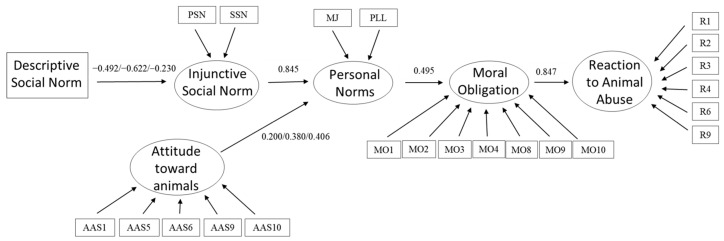
Final explanatory model of Reaction to animal abuse as a function of attitudes, norms and Moral obligation, as well as results of the structural equation analysis (where three values correspond to protected/pet/farm animals).

Finally, structural invariance was examined by adding equality constraints on all structural parameters across the three groups and comparing this model with the previous one (with constrained loadings and intercepts). The fully constrained model yielded adequate fit indices: χ^2^ (706) = 1088.7, *p* < 0.001, CFI = 0.917, TLI = 0.910, and RMSEA = 0.054, CI [0.047–0.060]. Structural invariance was achieved after freeing the structural parameters Personal Norm~Attitudes towards animals and Personal Norm~Descriptive Social Norm: Δχ^2^ (40) = 36.3, *p* > 0.05, ΔCFI = 0.001, ΔTLI = 0.007, and ΔRMSEA = 0.009. Therefore, some differences were observed in two structural parameters concerning the relationships of the latent variables Attitudes toward animals and Personal Norm and of Descriptive Social Norm and Injunctive Social Norm. The different loadings arising from releasing these two structural parameters depending on the category of animal are reflected in the final model shown in Figure 3.

The results obtained indicate that the proposed model has a good fit (RMSEA = 0.054, 90% CI = [0.047, 0.060]; CFI = 0.917; TLI = 0.904; NNFI = 0.910), although χ^2^ remains statistically significant (χ^2^ (706, *N* = 624) = 1088.432, *p* < 0.001). Furthermore, looking at R^2^ values, it can be concluded that, in all three cases, the predictive capacity of the models is very high, as the model explains 77% of the variance of the Reaction to Animal Abuse for the pets and farm animals groups and 72% for protected animals. However, there were differences in the percentage of variance explained by the other endogenous variables. The highest percentage of explained variance of Moral Obligation was found in the farm animals group (30%) compared to that of the protected animals (24%) and pets (10%) groups. The highest percentage of explained variance of Personal Norm was found in pets (98%), followed by protected animals (75%) and farm animals (63%). This is to the extent that the variable Attitudes toward animals has higher structural parameters in the case of farm animals (0.41) and pets (0.38) than in the case of protected animals (0.20). Finally, concerning the Social Injunctive Norm, the same pattern is followed as for Personal Norm, since the highest percentage of explained variance is found for pets (39%), followed by protected animals (24%) and farm animals (6%). At this point it is worth noting that, as was the case for Attitudes toward animals and Personal Norm, Descriptive Social Norm has lower structural parameters in relation to Injunctive Social Norm for farm animals (−0.23) than for protected animals (−0.49) and pets (−0.62).

## 4. Discussion

The aim of this study was to test a conceptual model that would allow us to explore the nature of the factors with the greatest explanatory power in relation to people’s reaction to animal abuse. The results obtained confirm that the moral obligation, understood as the feeling that one should act in some way, is the best predictor of the intention to intervene in the face of animal abuse, as expected. Moral obligation activates the personal norm insofar as it motivates people to act appropriately in response to the behavior one is trying to stop, which, in this case, is animal abuse [39]. These results are consistent with Schwartz’s [32] formulation and with previous evidence obtained with different types of prosocial and antisocial behavior [13,26].

### 4.1. Moral Obligation, Personal, and Social Norms

The result that the moral obligation acts by activating the personal norm is worth commenting on, insofar as few studies simultaneously analyze the impact of both variables (see [39]). Therefore, in the hypothesized model, it was originally proposed that this activation could occur through the different types of internal regulation, following Deci and Ryan’s [41] self-determination theory. However, the factor structure of introjected, identified, and integrated regulation was not confirmed. The analysis of the items used as indicators of each type of regulation showed a high multicollinearity that made it advisable, firstly, to eliminate identified regulation and, secondly, to unify integrated and introjected regulation into a single internal regulation.

Although these three types of regulation have been clearly defined theoretically, in some studies, participants have difficulties differentiating between them [30], especially regarding identified regulation which, in this case, shared the variance with both introjected and integrated regulation. This overlap between types of regulation may explain why the definition of the moral obligation in the model with the highest fit index was that of a single type of internal regulation. The different types of regulation can be clearly defined from a theoretical point of view, but their operationalization through indicators and the understanding of the differences between one type and another by non-academics is more complex. It may be that the problem lies in the wording of the items, which does not allow participants to detect the differences that are supposed to exist between the types of regulation. Also, the items used in the scientific literature on the subject are differentiated in linguistic terms with nuances (e.g., “it would be the most sensible thing to do” vs. “it is consistent with the way I am”) that may go unnoticed by those unfamiliar with Deci and Ryan’s [41] theory, even in academia. This limitation should be assessed in future research.

As far as the personal norm is concerned, the results indicate that the personal conviction that animal abuse is wrong is primarily defined as the perceived legitimacy of the law regulating the treatment of animals [47] but also as the negative moral evaluation of animal abuse [48]. The personal norm, although a conviction of the individual, is a social product insofar as it is the result of the processes of self-categorization [35] and identification [34] with a reference group [13]. For this reason, it was anticipated and verified that the personal norm about animal abuse has as its antecedent the injunctive social norm, understood as the perception of what the most important social referents, in the first place, and the rest of the people, in the second place, think about animal abuse. Using self-report measures as the sole evidence of this personal norm may lead to response bias, as suggested by the high scores obtained. The use of other measurement procedures, such as reaction time to an animal category stimulus, can help to estimate how internalized a norm against animal abuse is and to detect greater variability in responses.

The injunctive social norm is, in turn, influenced by the descriptive social norm, understood as the perception of what most people do. These results are consistent with Cialdini et al.’s [29] focal theory of normative conduct. This relationship between descriptive and injunctive social norms is stronger for pets than for protected and farm animals. This means that the more people are believed to abuse animals, the less negatively the abuse is perceived. As people have less contact with farm animals than with pets, it is reasonable to expect that they have less information about how other people treat them.

### 4.2. Attitudes Toward Animals

Another noteworthy finding concerns the contribution of attitudes toward animals to the explanation for the reaction to animal abuse. Until now, these attitudes have been linked to other attitudes, such as social dominance, or to behaviors that do not involve a direct human-animal relationship, such as choosing one dietary option or another [33]. Based on previous research (e.g., [21,22]), it was hypothesized that attitudes toward animals would influence behavior indirectly through the personal norm. The results obtained indicate that it is necessary to consider the category of animal when analyzing this relationship. The importance of attitudes toward animals in general, as antecedents of the personal norm, is greater for farm animals than for protected animals and pets. It stands to reason that one needs to have a very positive attitude toward animals to consider the abuse of farm animals as unacceptable, probably because it is more difficult to ignore the social acceptance of their instrumentality to humans [38]. This result may be of interest in the design of campaigns to raise awareness about animal abuse in farms and in businesses dedicated to animal consumption, promoting positive attitudes toward animals that are considered food, such as those shown frequently in childhood [38].

Initially, it was proposed to include in the model both attitudes toward animals, measured with the AAS [39], and speciesism, measured with the SS [50], assuming that these were two independent constructs. However, it was found that there was negative covariance between the two constructs, possibly as a result of redundancy in what the items of each scale measure. Therefore, in order to achieve an adequate fit of the model, it was decided to eliminate the speciesism measured with the SS, as it was the one with the least loading. This result supports the decision of authors such as Stoeber et al. [14] who opt for the AAS scale, instead of the SS, to measure speciesism.

### 4.3. Limitations of the Study

At this point, it must be acknowledged that the main limitation of this study is the lack of robustness of the Spanish adaptations of the AAS and SS when introduced into the model. Although the internal consistency values provided by Suárez-Yera et al. [51] and those calculated with the same data but independently of the model were acceptable, the collinearity of the items within the same scale and their relationship with those of the other scale compromised the fit of the model. To address this problem, future research should improve the psychometric properties of available instruments for measuring attitudes toward animals and speciesism or develop new ones that are invariant to culture. Another limitation that should be addressed in future work is the internal consistency of the two indicators chosen to measure the injunctive social norm. Although the factor loadings of both indicators in relation to the latent variable and their significance have been adequate, it is possible that adding more indicators that measure this variable could lead to better internal consistency and better model fit.

### 4.4. Contributions to Animal–Human Relationship Research

Despite its limitations, this study is a novel contribution to the research on the animal–human relationship research for several reasons. Firstly, it highlights the explanatory capacity of social and personal norms and their activation through moral obligation in relation to the reaction to animal abuse. At the same time, it raises the question of what other factors may be helping to explain the remaining variance of the moral obligation and the injunctive social norm, since the personal norm accounts for just over a third of the former and the descriptive social norm for just over a fifth of the latter.

Secondly, this study highlights that legislative changes do not have a significant influence on people unless they are internalized as personal convictions about the relationship between humans and animals. Certainly, the behavior under study has not been that of abusing an animal but of reacting to abuse. However, it seems reasonable to expect that, if the personal norm about animal abuse promotes a reaction to the abuse itself, it might also reduce the likelihood of that abuse taking place. Future research will tell whether or not this hypothesis is confirmed with respect to animal abuse, as has been found with other environmental crimes [26]. It also underlines the importance of informal social control over person behavior to reduce the prevalence of undesirable behavior toward animals. This means that it may be more effective to activate a sense of moral obligation among community members to defend and protect animals than to increase the number of law enforcement officers.

Finally, the results obtained show that the model tested is useful in explaining the reaction to animal abuse, whether they are protected species, pets, or raised for human consumption. However, they also suggest that the influence of attitudes toward animals on the personal norm, as well as of the descriptive social norm on the injunctive social norm, varies depending on the category of animal abused. This means that what people perceive others do influences to a greater or lesser extent what they consider socially acceptable or reprehensible, depending on whether the animal abused is, for example, a dog, a kestrel, or a chicken. Likewise, attitudes toward animals in general will have a greater influence on individuals’ convictions about whether it is wrong to abuse one or another of these animals. Future research should therefore delve deeper into how people’s relationships with animals are mediated by the role played in their categorization not only by their warmth and competence [62], attractiveness [63], or emotional proximity [1] but also by the instrumentality attributed to them socially and culturally [38].

## 5. Conclusions

The results of this study show that the moral obligation, understood as the feeling that one should act in some way, is the best predictor of the intention to intervene in cases of animal abuse. The moral obligation activates the personal norm insofar as it motivates one to act appropriately in response to the behavior one is trying to stop, which is, in this case, animal abuse. The personal norm that animal abuse is wrong is primarily defined as the perceived legitimacy of the law regulating the treatment of animals, but also as the negative moral evaluation of animal abuse. Although a conviction of the individual, the personal norm is a social product insofar as it is the result of the processes of self-categorization and identification with a reference group. For this reason, the personal norm about animal abuse has as its antecedent the injunctive social norm, understood as the perception of, first, what the most important social referents think about animal abuse, and, second, what the rest of the people think this issue.

Laws are injunctive social norms codified in written statements that may reflect social beliefs but also specific political and economic interests. Therefore, legislative changes do not have a significant effect on individuals if they are not internalized as personal convictions about the human–animal relationship. These findings also underline the importance of informal social control in reducing the prevalence of undesirable behavior toward animals, indicating that it may be more effective to activate a sense of moral obligation among community members to defend and protect animals than to increase the number of law enforcement officers. The model tested is useful in explaining the reaction to animal abuse, whether they are protected species, pets, or raised for human consumption. However, the results also suggest that the influence of attitudes toward animals on the personal norm and of the descriptive social norm on the injunctive social norm varies depending on the category of animal abused. Future research should therefore delve deeper into how people’s relationships with animals are mediated by the role played in their categorization not only by their warmth and competence, attractiveness, or emotional proximity, but also by the instrumentality attributed to them socially and culturally.

## Figures and Tables

**Table 1:** Descriptive statistics of the indicators included final explanatory model of Reaction to Animal Abuse as a function of attitudes, norms and Moral Obligation.

	*M*	*SE*	*SD*	Skewness	Kurtosis
DSN	2.133	0.0982	2.45	1.200	0.7475
PSN	7.795	0.0942	2.35	−1.348	1.5357
SSN	9.101	0.0801	2.00	−3.052	9.6021
MJ	9.708	0.0492	1.23	−5.735	36.3516
PLL	9.550	0.0520	1.30	−3.962	18.9455
AAS1	7.785	0.1386	3.46	−1.376	0.3407
AAS5	7.837	0.1199	2.99	−1.425	0.9842
AAS6	8.024	0.1108	2.77	−1.420	1.1704
AAS9	7.298	0.1437	3.59	−1.072	−0.3583
AAS10	7.191	0.1405	3.51	−0.980	−0.4936
MO1	7.877	0.1086	2.71	−1.352	1.0547
MO2	8.795	0.0812	2.03	−2.091	4.4476
MO3	8.830	0.0811	2.03	−2.179	4.7577
MO4	8.279	0.1021	2.55	−1.644	1.9834
MO8	7.904	0.1018	2.54	−1.261	0.9368
MO9	8.510	0.0810	2.02	−1.545	2.0782
MO10	8.567	0.0798	1.99	−1.612	2.4705
R1	6.396	0.1281	3.20	−0.524	−0.8964
R2	7.942	0.1084	2.71	−1.401	1.1913
R3	7.567	0.1093	2.73	−1.079	0.3434
R4	8.167	0.1032	2.58	−1.612	1.9753
R6	6.748	0.1313	3.28	−0.709	−0.7535
R9 *	8.322	0.0943	2.35	−1.591	1.9320

Note. * Responses to this indicator were reversed before statistical analyses were performed, since their direction is opposite to the rest of the items in the scale.

**Table 2:** Standardized factor loadings and significance of the observable indicators of each latent variable and associated ω values.

	Latent Variables			
Observable Variables	Estimate	*z*	*SE*	*SD*
Injunctive Social Norm (ω = 0.42)				
PSN	1			0.459
SSN	1.277	7.069 ***	0.181	0.623
Personal Norm (ω = 0.71)				
MJ	1			0.591
PLL	1.074	11.475 ***	0.094	0.806
Attitudes toward animals (ω = 0.71)				
AAS1	1			0.529
AAS5	1.014	7.622 ***	0.133	0.557
AAS6	1.045	7.683 ***	0.136	0.652
AAS9	1.097	7.299 ***	0.150	0.565
AAS10	1.007	6.933 ***	0.145	0.519
Moral Obligation (ω = 0.88)				
MO1	1			0.55
MO2	0.619	10.083 ***	0.061	0.469
MO3	0.891	16.826 ***	0.053	0.689
MO4	0.981	15.743 ***	0.062	0.566
MO8	1.249	17.725 ***	0.07	0.777
MO9	1.023	17.417 ***	0.059	0.824
MO10	0.977	15.583 ***	0.063	0.802
Reaction to Animal Abuse (ω = 0.78)				
R1	1			0.518
R2	0.812	13.000 ***	0.062	0.488
R3	0.758	11.084 ***	0.068	0.486
R4	0.694	9.720 ***	0.071	0.489
R6	1.009	14.451 ***	0.070	0.520
R9 *	0.674	10.807 ***	0.062	0.454

Notes. * Responses to this indicator were reversed before statistical analyses were performed, since their direction is opposite to the rest of the items in the scale; *** *p* < 0.001.

## Data Availability

The data presented in this study are openly available in OSF at https://osf.io/v7jsx (accessed on 13 November 2025), reference number v7jsx.

## Appendix A

Items used as indicators in the final model of the reaction to animal abuse as a function of attitudes, norms, and moral obligation (English translation and Spanish original wording).

Social and Personal Norms

Descriptive Social Norm (DSN). Most people treat animals this way//La mayoría de la gente trata así a los animales [29].

Subjective Social Norm (SSN). The people I care about most think that treating animals this way is wrong//Las personas que más me importan piensan que tratar así a los animales está mal [30,31].

Prescriptive Social Norm (PSN). Most people think that treating animals like that is wrong//La mayoría de la gente piensa que tratar así a los animales [29].

Moral Judgment (MJ). I personally think that treating animals like that is wrong//Yo personalmente creo que tratar así a los animales está mal [31].

Perceived Legitimacy of the Law (PPL). It is good that there are laws regulating the treatment of animals//Está bien que haya leyes que regulen el trato hacia los animales [47,48].

Moral Obligation

These items were taken from the original English version of the Motivation Toward the Environment Scale [42], which is based on Deci and Ryan’s [41] self-determination theory. The Spanish wording came from Martín et al.’s [46] Spanish adaptation of this scale. Item MO1 was from Schwartz’s [32].

MO1. I would feel obliged to act in some way//Me sentiría obligado/a a actuar de algún modo.

MO2. I would feel proud to act in some way//Me sentiría orgulloso/a de actuar de algún modo.

MO3. If I did not act in some way, I would feel guilty//Si no actuara de algún modo me sentiría culpable.

MO4. If I did not act in some way, I would feel ashamed//Si no actuara de algún modo me sentiría avergonzado/a.

MO5. To act in some way would be the most reasonable thing to do//Actuar de algún modo sería lo más sensato.

MO6. Acting in any way would be important to me//Actuar de algún modo sería importante para mí.

MO7. I personally value acting in any way//Personalmente valoro actuar de algún modo.

MO8. I cannot imagine myself without acting in some way//No me imagino a mí mismo/a sin actuar de algún modo.

MO9. Acting in some way is consistent with the way I am//Actuar de algún modo es coherente con mi forma de ser.

MO10. Acting in some way fits in with my outlook on life//Actuar de algún modo encaja con mi forma de ver la vida.

Reactions to Animal Abuse

R1. I would stand between the animal and the abuser//Me interpondría entre el animal y la persona que agrede.

R2. I would shout at the abuser to stop//Le gritaría a la persona que agrede que parara.

R3. Alert other people nearby//Alertaría a otras personas que estuvieran cerca.

R4. I would call 112, the local police or the Nature Conservation Service//Llamaría al 112, a la policía local o al SEPRONA.

R5. I would record it with my mobile phone//Lo grabaría con el móvil.

R6. I would file a report//Pondría una denuncia.

R7. I would throw something at the offender//Le tiraría algo a la persona que agrede.

R8. I would stop to see what is happening//Me pararía a ver lo que está pasando.

R9. I would go on my way//Seguiría mi camino.

Animal Attitude Scale

Spanish adaptation by Suárez-Yera et al. [51] and original English wording by Caviola et al. [50].

AAS1. It is morally wrong to hunt wild animals just for sport//Cazar animales salvajes solo por deporte es inmoral.

AAS2. El uso de animales en la investigación médica es correcto//I do not think that there is anything wrong with using animals in medical research.

AAS3. Criar ganado para el consumo humano es acceptable//I think it is perfectly acceptable for cattle and hogs to be raised for human consumption.

AAS4. Los humanos tienen el derecho de utilizar a los animales para lo que crean conveniente//Basically, humans have the right to use animals as we see fit.

AAS5. The slaughter of whales and dolphins should be immediately stopped even if it means some people will be put out of work//La matanza de mamíferos marinos (ballenas, delfines, etc.) debe parar, aunque eso implique el desempleo de muchas personas.

AAS6. I sometimes get upset when I see wild animals in cages at zoos//Es molesto ver animales salvajes enjaulados en los zoológicos.

AAS7. La cría de animales para obtener sus pieles está justificada//Breeding animals for their skins is a legitimate use of animals.

AAS8. Si queremos saber sobre la biología, no hay más remedio que diseccionar animales//Some aspects of biology can only be learned through dissecting preserved animals such as cats.

AAS9. It is unethical to breed purebred dogs for pets when millions of dogs are killed in animal shelters each year//Es inmoral criar perros de raza para venderlos como mascotas cuando millones son asesinados cada año en los refugios.

AAS10. The use of animals such as rabbits for testing the safety of cosmetics and household products is unnecessary and should be stopped//El uso de animales para probar la seguridad de los cosméticos y los productos domésticos es innecesario y debe detenerse.
